# The Management of Cardiometabolic Risk in MAFLD: Therapeutic Strategies to Modulate Deranged Metabolism and Cholesterol Levels

**DOI:** 10.3390/medicina61030387

**Published:** 2025-02-23

**Authors:** Annalisa Pezzoli, Ludovico Abenavoli, Marialaura Scarcella, Carlo Rasetti, Gianluca Svegliati Baroni, Jan Tack, Emidio Scarpellini

**Affiliations:** 1 Internal Medicine Unit, “ Madonna del Soccorso” General Hospital, 63074 San Benedetto del Tronto, Italy; annalisa.pezzoli@sanita.marche.it (A.P.); carlo.rasetti@sanita.marche.it (C.R.); 2 Department of Health Sciences, University “Magna Graecia”, 88100 Catanzaro, Italy; l.abenavoli@unicz.it; 3 Anesthesia, Intensive Care and Nutritional Science, Azienda Ospedaliera “Santa Maria”, Via Tristano di Joannuccio, 05100 Terni, Italy; m.scarcella@aospterni.it; 4 “Hepatic Damage and Transplantation “ Unit, Polytechnics University of Marche, 60121 Ancona, Italy; gsvegliati@staff.univpm.it; 5 Translational Research in Gastroeintestinal Disorders, Gasthuisberg University Hospital, KULeuven, Herestraat 49, 3000 Leuven, Belgium; jan.tack@med.kuleuven.be

**Keywords:** fatty liver, NAFLD, MAFLD, dyslipidemia, diet, statin, PCSK9, nutraceuticals, bempedoic acid

## Abstract

*Background and Objectives:* Fatty Liver Disease is a major health problem worldwide. We can distinguish liver steatosis as non-associated or associated with chronic/acute alcohol consumption. These two entities share similar stages ranging from hepatic fat storage (namely, steatosis) to inflammation, necrosis, and fibrosis until hepatocellular carcinoma (HCC). Over time, “Metabolic Associated Fatty Liver Disease” (MAFLD) has replaced nonalcoholic fatty liver disease (NAFLD) nomenclature and has included cardiometabolic criteria in these patients definition. Thus, obesity, type 2 diabetes mellitus (T2DM), hypertension, and dyslipidemia are MAFLD features and are of the metabolic syndrome. Importantly, there is not a specific treatment for MAFLD, but there are therapeutic strategies that act on metabolic dysfunction related to MAFLD. They can reduce the progression of liver fibrosis and its complications. *Materials and Methods:* For all these reasons, we conducted a narrative review of the literature, and we focused on metabolic dysfunction related to MAFLD, with a special regard for cholesterol metabolism. *Results:* MAFLD is a recently redefined condition that better describes the metabolism derangement responsible for fatty liver disease. This distinguishes MAFLD from NAFLD. In fact, the diagnostic criteria for MAFLD require the presence of liver steatosis together with at least one of the following: obesity, T2DM, or evidence of metabolic disorder such as hypertriglyceridemia, low high-density lipoprotein cholesterol, or hypertension. As a result, MAFLD is closely linked to an increased cardiometabolic risk. Current therapeutic approaches can be used to reduce this risk, focusing on lifestyle interventions and pharmacological strategies. Several treatments in patients diagnosed with MAFLD are mainly cholesterol-lowering remedies. Among these, Pro-protein Convertase Subtilisin/Kexin type 9 inhibitors (PCSK9i) show the most promising efficacy profile but data on liver fibrosis are lacking. Agonists of GLP-1 receptor, Sodium-glucose cotransporter-2 inhibitors (SGLT2i) and Dipeptidyl Peptidase-4 inhibitors (DPP-4i) have a “ multi-hit “ action allowing their use also in diabetic patients with MAFLD. *Conclusions:* Lifestyle modifications, some nutraceuticals, statins, incretins, and PCSK9i have changed the natural course and significantly improved the cardiometabolic outcomes of MAFLD. Emerging cholesterol-lowering drugs, such as Bempedoic acid, can overcome low compliance to statins’ use and their controversial effect on liver fibrosis. Finally, medications targeting insulin resistance allow for strategic interventions of the convoluted pathophysiology of MAFLD in multiple steps, with the potential to reduce liver steatosis, inflammation, and necrosis and, sometimes even to reverse liver fibrosis.

## 1. Introduction

**Figure 1:**
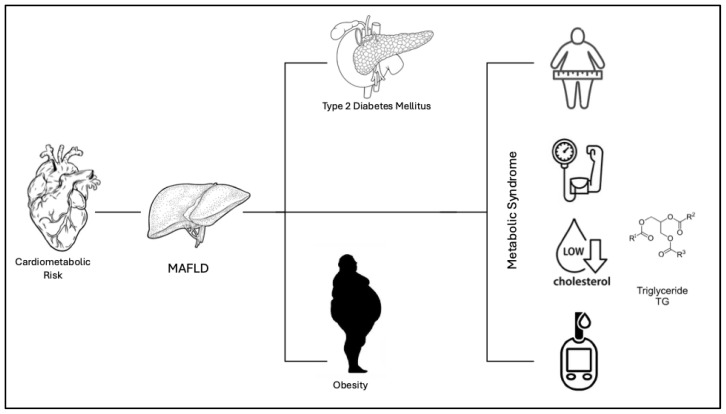
The Figure shows the link between cardiometabolic risk and MAFLD. According to the definition of “Metabolic Associated Fatty Liver Disease”, MAFLD, the disease can present with various metabolic conditions including obesity, insulin resistance (IR), type 2 diabetes mellitus (T2DM), hypertension, hyper-lipidemia, and metabolic syndrome.

Fatty liver disease is a rising health problem. It was firstly classified as nonalcoholic fatty liver disease (NAFLD) or alcohol-related fatty liver disease (AFLD) depending on the absence/presence of alcohol use/abuse. In the last two decades, NAFLD prevalence (ranging from 12 to 22% of the general population) has been associated with various metabolism alterations (e.g., obesity, insulin resistance (IR), type 2 diabetes mellitus (T2DM), hypertension, hyperlipidemia and, more comprehensively, metabolic syndrome). Consequently, NALFD has been renamed as metabolic-associated fatty liver disease, MAFLD [1]. Obesity, diabetes, and Metabolic Syndrome (MetS) are key drivers of liver fat deposition in ’Metabolic Associated Fatty Liver Disease’. Therefore, MAFLD diagnosis is reached according to liver fat deposition detection (via radiology imaging, liver biopsy, or, more recently, blood biomarkers) and one of three major dysmetabolic conditions: obesity or being overweight, type-2 diabetes mellitus, and the presence of two or more metabolic abnormalities [2,3]. Thus, MAFLD prevalence has risen over that of NAFLD, overcoming 30% of the general population [2,3]. Robust evidence points out a strong association between increased cardiovascular risk and MAFLD. Atherosclerotic carotid plaques and a fatty liver are a common finding at baseline patients’ evaluation [4] [Figure 1].

Indeed, the MAFLD definition has drawbacks: patients with fatty liver are not diagnosed according to the amount and frequency of alcohol use/abuse. Further, a vast group of etiologies are included in the definition. For these reasons, the new “metabolic associated steatotic liver disease”, MASLD, requires at least one out of five cardiometabolic risk factors and distinguishes between alcohol use or abuse. Thus, the term “metabolic and alcohol-related/associated liver disease” (MetALD) has been introduced to describe patients with MASLD who consume greater amounts of alcohol per week (140–350 g/week and 210–420 g/week for females and males, respectively) [5]. In addition, the MASLD definition overcomes the stigma of the term “fatty” and, more importantly, includes the pathophysiologic dysmetabolic milieu of the disease [6,7].

MASLD prevalence reaches almost 25% of the global population with a growing perspective [6]. This prevalence follows that of obesity and is going to be the main contributor of increased prevalence of chronic hepatic diseases and hepatocellular carcinoma worldwide [7].

The age range for MAFLD patients is between 40 and 60 years. Indeed, the disease can be diagnosed also in children older than 10 years. MAFLD seems to significantly affect more males than females, perhaps at a younger age. This sex-based distinction is the opposite for patients older than 65 years [8]. This condition often progresses from simple fatty liver to more serious stages like steatohepatitis (NASH) and cirrhosis, with or without hepatocellular carcinoma. However, the transition from liver steatosis is not linear and sometimes steatohepatitis patients can develop hepatocellular carcinoma (HCC) without cirrhotic degeneration [9]. Given the increasing number of cases of MAFLD, several therapeutic strategies have been studied to act on metabolic dysfunctions affecting liver disease. The latter are centered on lifestyle modifications. Indeed, there are pharmacological interventions that address the shared underlying metabolic and hepatic pathologic pathways. The aim of these strategies is to focus on the most common metabolic disorders linked to MAFLD, such as: insulin resistance (often treated with metformin, thiazolidinediones), oxidative stress (counteracted with vitamin E, pentoxifylline), and inflammatory cytokines (modulated via anti-TNF-a, TGF-β, IL-11) [10]. Indeed, lowering cholesterol levels (with statins, ezetimibe or their combination, bempedoic acid and, due to statin-intolerance, the novel proprotein convertase subtilisin/kexin 9 inhibitors) is a prominent target.

Thus, we performed a narrative review of the literature’s evidence on the definition of MAFLD, its diagnosis, the role of lipogenesis/hepatic lipid deposition and insulin resistance in its pathophysiology, and the relevance of remedies and drugs positively affecting deranged metabolism (e.g., lowering cholesterol and affecting insulin resistance). We have chosen the MAFLD and NAFLD nomenclature as it accounts for most of the reviewed studies.

## 2. Materials and Methods

We made a search on PubMed, Medline for the literature data (namely, original articles, reviews, meta-analyses, and case series) using the following keywords, their acronyms, and their associations (e.g., “and”): “Fatty liver”, “NAFLD”, “MAFLD”, “Dyslipidemia”, “Diet”, “PCSK9”, “Statin”, “Nutraceutical Therapies”, “Bempedoic acid”. Importantly, we chose the MAFLD definition because most of the literature studies retrieved were performed in patients with this nomenclature of disease. We considered articles in the timeframe of 2000–2024 years. In these years, the terms NAFLD and MAFLD were the most frequently used. We selected articles published in English and involving human and animal models of NAFLD and MAFLD. The MASLD definition and physiopathologic data were included in the Introduction section to complete the NAFLD and MAFLD pathophysiology description.

## 3. Results

### 3.1. Metabolic Dysfunction-Associated Fatty Liver Disease (MAFLD)

#### 3.1.1. Diagnostic Criteria for MAFLD

According to the most recent guidelines, NAFLD could be considered, according to liver fat accumulation detected at abdominal magnetic resonance imaging (MRI) and/or biopsy in the absence of other hepatic injury causes (e.g., alcohol, hepatotoxic drugs, toxins, viral infections, primary liver disease) [11,12]. Hepatic steatosis can be first diagnosed by ultrasound when the hepatic parenchyma shows supranormal brightness [12]. Indeed, liver steatosis can be underdiagnosed or missed when fat deposition spares more than 67% of hepatic parenchyma [13]. In addition, the term NAFLD has been replaced by MAFLD because it only describes liver fat accumulation, does not mention alcohol use/abuse, and does not rely on metabolism alterations. Thus, experts in the field have performed accurate consensus conferences to modify the definition. In particular, the MAFLD term was proposed in 2020 and connected the diagnosis of fatty liver disease and one of the following: Type 2 Diabetes Mellitus (T2DM), obesity, and metabolic dysfunction. Therefore, the stigma of “non-alcoholic “was removed [13].

To provide further detail and practically, liver steatosis must be accompanied by at least two dysmetabolic findings [13]: waist circumference ≥ 102/88 cm in Caucasian men/women or ≥ 90/80 cm in Asian men/women; blood pressure ≥ 130/85 mmHg or antihypertensive drugs; plasma triglycerides (TG) ≥ 150 mg/dL or TG lowering drugs; plasma high-density lipoprotein cholesterol (HDL-C) < 40 mg/dL for men and <50 mg/dL for women or lipid-lowering drugs; fasting plasma glucose (levels 100 and 125 mg/dL or 2 h post-load); glucose levels (140–199 mg/dL) or glycosylated hemoglobin (HbA1c) between 5.7 and 6.4%, Homa index score ≥ 2.5; and high- sensitivity C-reactive protein levels > 2 mg/L [Table 1].

Data on the relationship between sex, race, and other socioeconomic factors and liver steatosis are available mainly for NALFD. Indeed, there are substantial disparities in the development of NAFLD according to race and ethnicity because of genetics and environmental and social factors. In detail, the prevalence of NAFLD in the US population appears to be higher among Hispanics, followed by non-Hispanic Whites and Asians, and lastly, African Americans [14]. Similarly, the same disparity has also been observed in hospitalized patients [15]. Interestingly, Japanese Americans have a greater risk for NAFLD development because of high visceral adiposity prevalence [16]. Finally, Hispanics appear to have a higher NAFLD prevalence, an earlier onset, and a worse metabolic profile vs. other ethnicities. Unfortunately, data reported come almost exclusively from studies run within the US population [17].

The impact of socioeconomic status on NAFLD prevalence is varied around the world. Initially, NAFLD prevalence seemed to be higher among individuals with lower socioeconomic status in Western countries [18]. Similarly, South Korea data found people with a low socioeconomic status having a significantly higher risk of developing NAFLD (OR 1.7) [19]. Oppositely, a Chinese study found people with a higher median income having a two-fold higher risk of developing NAFLD vs. the low-income subjects [20]. Such disparities are linked to food security and food composition. Almost 30% of US adults with low-income have NAFLD and live in food-insecure households [18]. Conversely, the prevalence of food insecurity is much higher among the Iranian NAFLD population (56.8%) vs. non-NAFLD subjects (26.1%) [21].

#### 3.1.2. Clinical and Laboratory Indexes for Steatosis Monitoring

Both NAFLD and MAFLD need an accurate grading and staging of steatosis activity and fibrosis. The grading and staging describe disease progression and allow physicians to predict patient outcomes and select the best therapeutic option. Thus, laboratory indexes and non-invasive scoring systems have emerged as valuable tools in this context. The disease is considered a continuum of stages from liver steatosis to steatohepatitis (namely, NASH in the case of NAFLD or Metabolic Dysfunction-Associated Steatohepatitis (MASH), in the case of MAFLD). In fact, disease activity and fibrosis can be evaluated through non-invasive methods or liver biopsy (e.g., NAFLD Activity Score and degree of fibrosis, respectively). In one regard, laboratory tests support the MAFLD diagnosis and allow the evaluation of dysmetabolic conditions associated with hepatic steatosis evolution. However, biomarkers used for liver fibrosis detection typically indicate matrix turnover, but not the extent of extracellular matrix deposition. Moreover, no biomarker is specific for liver fibrosis detection. In fact, extra-hepatic inflammatory and oxidative states can contribute to fibrosis development in MAFLD. There are two types of markers that determine fibrosis level in the liver: indirect and direct ones [Table 2].

The indirect markers describe deranged hepatic metabolism: aspartate amino Transferase (AST), Alanine amino Transferase (ALT), platelet count, Gamma-Glutamyl Transferase (GGT), total bilirubin, alpha 2-macroglobulin, or alpha 2-globulin (mainly haptoglobin). However, they do not help to detect the presence of fibrosis. Therefore, scores built from a combination of multiple biomarkers can have a higher diagnostic accuracy [28,29].

On the other hand, direct markers can help predict the presence of liver fibrosis. In fact, they are biomarkers of collagen synthesis/degradation, extracellular matrix glycoproteins, proteoglycans, and glycosaminoglycans (PIIINP: amino-terminal Propeptide of type III Procollagen; TIMP-1: Tissue Inhibitor of Metalloproteinase; TNF: Tumor Necrosis Factor; MMP: Matrix Metallo Proteinase). Furthermore, other biomarkers are pro-inflammatory molecules, such as Transforming Growth Factor beta-1 (TGF-β1), Insulin-Like Growth Factor (IGF-1), and endothelin-1 and inflammatory mediators such as C-Reactive Protein (CRP), Interleukin (IL)-6, and pro-coagulant factors such as fibrinogen, factor VIII, and plasminogen activator inhibitor-1 [29,30,31].

Finally, there are also scores that can assist physicians in evaluating the progression and the degree of hepatic steatosis, such as the Hepatic Steatosis Index (HIS) [32], Fatty Liver Index (FLI) [33], the FIB-4 index [34], ELF test [Lee J], and APRI [34,35].

#### 3.1.3. Non-Invasive Imaging Techniques for MAFLD Diagnosis and Histological Findings

Several imaging techniques are currently employed to diagnose and assess the severity of MAFLD. Ultrasound (US) is the most used imaging available in current clinical practice due to its low cost and widespread availability. However, it has low sensitivity for liver steatosis detection and is not able to discriminate between liver steatosis and fibrosis [31]. Indeed, liver steatosis can be also frequently detected by computed tomography (CT) or MRI [36].

Alternatively, the controlled attenuation parameter (CAP) measured during elastography is a more sensitive radiologic tool, and the proton magnetic resonance spectroscopy (1H-MRS) is also an acceptable quantitative marker of steatosis. These measurements can be combined with laboratory biochemical testing in high-risk populations [37]. Indeed, the main European and American liver disease scientific societies (namely, EASL and AASLD) recommend the use of abdominal ultrasound and liver enzymes testing for all patients with documented metabolic risk factors [38]. CT is a more accurate diagnostic tool than ultrasound. However, its use is limited to mild steatosis patients because it requires radiation exposure [39]. While CT is more sensitive than ultrasound for evaluating hepatic fat content, substances like iron can interfere and impact the diagnosis [40]. The CAP technique, an ultrasound-based approach, measures steatosis (greater than 10%) but has been shown to be somewhat unreliable, even though it is still recommended in Asia-Pacific guidelines as a useful tool for NAFLD/MAFLD patients. MRI and magnetic resonance spectroscopy can detect liver fat and fibrosis, but their application in clinical practice is, as of yet, limited by the high costs. In detail, MRS has a complex protocol for routine clinical settings, despite its ability to detect 5.56% of liver fat content. Indeed, it can be considered the gold standard for diagnosing steatosis [41]. In recent years, transient elastography (Fibro Scan), an ultrasound-derived technique, has become more popular because it offers fast and convenient measurements of liver stiffness, which correlates closely with liver fibrosis stages [42]. It can be used either alone or in combination with a CAP measurement, giving a consensual liver stiffness and steatosis assessment [42].

Histological diagnosis remains the gold standard for confirming MAFLD and evaluating its severity, particularly in advanced stages. Liver biopsy is an invasive procedure with 0.05% risk of mortality linked to procedures’ complications. It allows the assessment of key histopathological features, including steatosis, inflammation, the ballooning of hepatocytes, and fibrosis [43]. Although biopsy has a high diagnostic accuracy, it is an invasive and costly protocol with limited routine application [31,44,45].

More interestingly, liver biopsy shows greater issues when applied to bigger populations because of the sampling error (namely, the steatosis/fibrosis-spared liver segment receiving the biopsy). The latter produces false-negative cases with misread prognosis.

Histologically, we distinguish macrovesicular and microvesicular steatosis.

In microvacuolar (or macrovesicular) steatosis, triglycerides commonly accumulate as a single large lipid vacuole relocating the nucleus at the periphery of the hepatocyte [46]. This histological feature is the most common finding and is typically retrieved in obese subjects [47], alcoholic liver disease, Wilson’s disease, and familial hypobetalipoproteinemia [47]. Usually, macrovacuolar steatosis is associated with hepatomegaly. In about 20% of cases, it may progress to steatohepatitis, with necroinflammation, ballooning degeneration of hepatocytes, and fibrosis [47]. It is important to acknowledge that fibrosis progresses to liver cirrhosis over weeks/months in drug-induced steatohepatitis. Indeed, obesity and other metabolic conditions favor cirrhosis development over decades [46].

The most common cause of microvesicular steatosis are drugs. Its clinical characteristics include liver failure, encephalopathy, multiorgan failure, and coma [47]. The rarer microvesicular steatosis recognizes the presence in the cytoplasm of several lipid droplets, which leave the nucleus at the center of the hepatocyte [46]. There is also a certain very rare association with macrovacuolar steatosis [46]. Small lipid droplets reflect severe mitochondrial dysfunction in the injured hepatocytes [48]. From a pathogenetic point of view, ATP deficiency has been linked to severe mitochondrial dysfunction and, altogether, these can lead to the growth of lipid droplets through the reduction of lipid synthesis or altered deranged expression of proteins and enzymes storing lipids [48]. In another hypothesis, triglyceride can be hydrolyzed in the largest lipid droplets to mobilize oxidable fatty acids [49]. Finally, lysosomal function derangement can favor small lipid droplets storage [49].

Other diseases with microvesicular steatosis are acute fatty liver of pregnancy, some inborn errors of mitochondrial fatty acids’ oxidation, and several mitochondrial cytopathies (i.e., genetic disorders of the OXPHOS system) [4,7,49]. Typically, Reye’s syndrome, triggered by an acute viral illness (e.g., influenza and varicella) and drugs (namely, the nonsteroidal anti-inflammatory drug (NSAID) aspirin (or herbal tea containing salicylate) and valproic acid) is associated with microvesicular steatosis [4,49].

In conclusion, non-invasive methods can be considered the first step for NAFLD/MAFLD diagnosis. Unfortunately, abdominal ultrasound has a significant inter-observer variability. Other techniques can result in over- or underestimation of the hepatic fibrosis stage [49]. When NASH/MASH and liver fibrosis are not diagnosed/detected by non-invasive methods, liver biopsy can be performed in individuals in whom the etiology of the liver disease needs to be clarified [44]. Non-invasive biomarkers and newer imaging techniques are becoming more and more popular for early patients’ evaluation.

### 3.2. Metabolic Dysfunction in MAFLD

#### 3.2.1. Lipid Metabolism and Insulin Resistance in MAFLD

**Figure 2:**
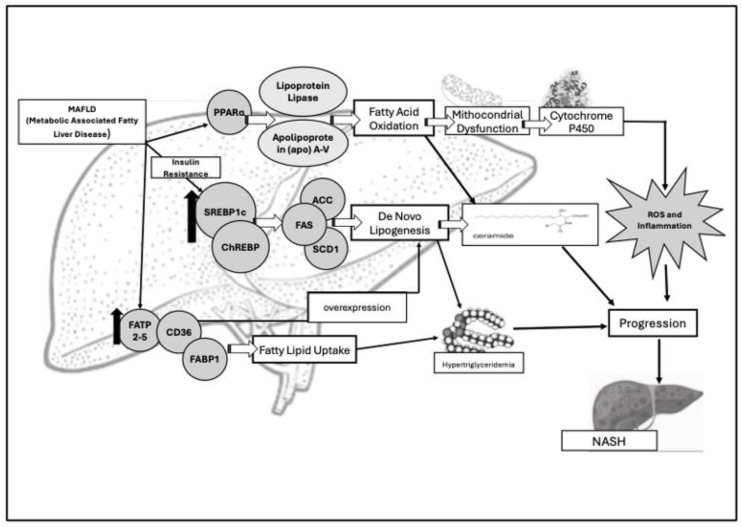
Main pathophysiologic key factors involved in deranged lipid metabolism and insulin resistance in MAFLD. Sterol regulatory element-binding protein 1c (SREBP1c) and fatty acid transport proteins (FATs) are upregulated (↑) in MAFLD patients and are responsible for deranged lipogenesis and lipid uptake. Lipogenesis is regulated by three enzymes: acetyl-CoA carboxylase (ACC), fatty acid synthase (FAS), and stearoyl-CoA desaturase-1 (SCD1). The result is the formation of ceramides (namely, palmitate, oleate, and palmitoleate), responsible for passage from liver steatosis to liver steatohepatitis (NASH/MASH). Their increased levels lead to hypertriglyceridemia. SREBP1c is primarily activated by insulin, especially in insulin resistance conditions. The latter impairs the suppression of lipolysis in adipose tissue, leading to an increased flux of free fatty acids (FFAs) up to the liver. Consensually, it has been determined that there is an increased expression of FA binding protein (FABP), FA transport protein (FATP), and CD36 genes responsible for fatty acid uptake and overexpression of de novo lipogenesis. FABP1 is specifically expressed in the liver where it transports fatty acids between organelles, binding cytotoxic free fatty acids, and aiding their oxidation or incorporation into triglycerides. The peroxisome proliferator-activated receptor-alfa (PPARα) regulates fatty acid oxidation across mitochondria, peroxisomes, and cytochrome pathways. PPARα activation induces the production of lipoprotein lipase (LPL) and Apolipoprotein (apo) A-V.

MAFLD physiopathology encompasses a complex interplay of lipid metabolism pathways. The most advanced form of liver fibrosis, MASH and, more specifically MASH-cirrhosis, is initiated by lipotoxicity. The latter is characterized by cellular inflammation, oxidative stress, and hepatocellular ballooning. MASH can irreversibly progress to liver cirrhosis and/or to the development of HCC. MASH progresses to cirrhosis and/or HCC due to several pathologic processes: cellular senescence, oxidative stress, autophagy, and ferroptosis [50,51]. In detail, hepatic fat accumulation and steatosis are a result of excessive lipid uptake, de novo lipogenesis, impaired oxidation of fatty acids, and dysfunctional lipid export [51,52] [Figure 2].

We must note that lipid metabolism plays a pivotal role in several crucial activities in humans. They include energy storage and release, cell membrane formation, hormone synthesis and transport, liposolubility of nutrients, and the regulation of inflammatory response [53]. MAFLD patients show their derangement.

Key molecular players, such as sterol regulatory element-binding protein 1c (SREBP1c) and fatty acid transport proteins (FATs), are upregulated in MAFLD, driving both lipogenesis and lipid uptake [50,52]. The liver’s de novo lipogenesis is regulated by acetyl-CoA carboxylase (ACC), fatty acid synthase (FAS), and stearoyl-CoA desaturase-1 (SCD1). Their machinery results in the formation of palmitate, oleate, and palmitoleate whose storage ends up in hypertriglyceridemia and liver steatosis [54]. The two transcription factors that regulate the enzymes (precisely, FAS, and SCD1), are SREBP1c and the carbohydrate regulatory element-binding protein (ChREBP). As a master regulator of the de novo lipogenesis pathway, SREBP1c is primarily activated by insulin and shows a significant increase in MAFLD patients vs. healthy individuals [44]. Moreover, the overexpression of SREBP-1c is linked to the upregulation of key enzymes of de novo lipogenesis and results in hepatic lipid accumulation [55]. Indeed, mice with MAFLD undergoing single-cell RNA sequencing (scRNA-seq) and computational network analyses to assess lipid signatures showed that high SREBP1 expression is not predictive of liver lipids’ accumulation. Further, the constitutive androstane receptor (CAR) is a key regulator of functional modules associated with cholesterol homeostasis, bile acid metabolism, fatty acid metabolism, and estrogens’ response. Thus, there is a significant correlation of its activation with steatohepatitis development in humans [56]. In addition, among the other enzymes regulated by SREBP1c, the isoforms of ACC have a significantly increased expression in MAFLD patients vs. controls [57,58]. Interestingly, MAFLD patients also exhibited altered expressions the of FA binding protein (FABP), FA transport protein (FATP) [57,58], and cluster of differentiation 36 (CD36) genes. The latter are responsible for fatty acid uptake and the overexpression of de novo lipogenesis [56]. Fatty Acid Binding Protein 1 (FABP1) is specifically expressed within the liver and transports fatty acids between organelles, binding cytotoxic free fatty acids. The protein is also involved in their oxidation/incorporation into triglycerides [58]. In fact, knockout FABP1 mice showed a reduced response to fasting-induced increases in hepatic triglyceride uptake and oxidation [54]. In addition, hepatic lipid uptake is regulated by FATPs and CD36, and FATP isoforms 2 and 5 are the most abundant in the liver [59]. Increased FATP5 expression in humans significantly correlates with higher hepatic steatosis in male MAFLD patients [60,61]. Moreover, hepatic CD36 protein levels’ expression rise under high-fat diet [62]. These findings suggest a connection between FATP5, CD36, and hepatic lipotoxicity [Figure 2].

De novo lipogenesis overexpression can lead to fat storage in MAFLD and, also to storage of toxic lipid species (e.g., ceramides), critical for liver fibrosis development.

The impaired oxidation of fatty acids has been detected within mitochondria in NAFLD research models. Very long-chain fatty acids are initially oxidized in peroxisomes before being further processed. Under conditions of lipid overload (e.g., high-fat diet), an alternative omega-oxidation pathway mediated by cytochrome P450 enzymes becomes active. This fuels fatty acid oxidation. Unfortunately, this pathway produces significant amount of reactive oxygen species (ROS) that trigger the inflammatory response and favor progression to NASH. The peroxisome proliferator-activated receptor-alfa (PPARα) plays a central role in regulating fatty acid oxidation across mitochondria, peroxisomes, and cytochrome pathways. PPARα modulates lipid and lipoprotein metabolism. It regulates the transcription of genes involved in the metabolism of triglycerides (TG)-rich lipoproteins and HDL [63]. Thus, PPARα activation induces lipoprotein lipase (LPL) and Apolipoprotein (apo) A-V synthesis expression. Conversely, its decrease is followed by apo C-III activation. This results in LPL activity inhibition and enhanced beta (β)-oxidation genes expressions [64] [Figure 2].

Current data from the literature demonstrate that also microRNAs (miRNAs) might be involved in NAFLD and MAFLD development. In detail, NAFLD animal models show miRNAs linked to deranged cholesterol metabolism and NAFLD [32,45,49]. Further, a comprehensive review of the literature including 19 articles demonstrated that 13 different miRNAs are related with the altered lipid metabolism typical of MAFLD. The most studied is miR122, one of the most abundant in the liver [57].

Insulin resistance (IR) is a central driver in the pathogenesis of MAFLD. It links hepatic steatosis to systemic metabolic dysregulation. IR impairs lipolysis inhibition within the adipose tissue, leading to an increased flux of FFAs up to the liver. The flux promotes hepatic triglyceride accumulation, exacerbating steatosis and triggering lipotoxicity. IR reduces hepatic glycogen synthesis and, on the other hand, enhances de novo lipogenesis via upregulation of transcription factors such as SREBP1c [65]. This imbalance is reinforced by oxidative stress, mitochondrial dysfunction, and chronic low-grade inflammation. Altogether, these processes create a vicious cycle that accelerates MAFLD progression toward MASH. Consensually, it has been determined that oxidative stress from mitochondrial dysfunction and reactive oxygen species reinforces inflammation and insulin resistance. IR downregulates lipases functioning and this results in the altered flow of fatty acids and of the intestinal production of chylomicrons (CM) and of hepatic very low-density lipoproteins (VLDL). Moreover, hyperinsulinemia increases fatty acid esterification and inhibits beta-oxidation that regulates triglycerides formation in liver [66]. In addition, dysmetabolic patients show increased oxidative stress and increased blood levels of glucose and lipoproteins, ending in foam cell formation and atherosclerotic disease [31].

Finally, the physiopathologic “multi-hit” hypothesis that integrates these pathways emphasizes the roles of insulin resistance, dyslipidemia, and inflammation in MAFLD pathogenesis, making lipid metabolism a crucial target for therapeutic strategies [67].

#### 3.2.2. Cardiovascular Risk in MAFLD: The Link Among Physiopathology and Clinical Features

MAFLD has been significantly associated with cardiovascular disease-related mortality. In fact, it is a major risk factor for cardiovascular diseases (CVD) like myocardial infarction, stroke, and heart failure [51]. This is supported by meta-analysis and systematic review data [68]. Moreover, MAFLD contributes to an accelerated progression of coronary atherosclerosis, heart failure, and arrhythmia [68,69]. Similarly, MASLD shows a similar risk profile for CVD [70]. In a large study involving over 8.8 million South Korean adults, MAFLD was significantly associated with a higher incidence of cardiovascular events [69,71].

### 3.3. Current Therapeutic Strategies Targeting Metabolism in MAFLD

#### 3.3.1. Diet and Lifestyle in the Treatment of MAFLD

To date, lifestyle interventions, particularly diet and physical activity, are the cornerstone for managing MAFLD. We aim to counteract the lifestyle changes derived from the rapid economic growth of the last 40 years. Specifically, a Westernized world has led to more meat and egg consumption. Conversely, ingestion of fruits, vegetables, and whole grains has decreased [72]. This alimentary shift is high in cholesterol [73]. Thus, a comprehensive approach addressing weight reduction, dietary changes, and increased physical activity is essential for improving liver health, metabolic dysfunction, and cardiovascular outcomes in MAFLD patients [74]. For example, overweight and obese MAFLD patients obtaining a weight loss of 7–10% show a decrease of hepatic steatosis grading and, of vascular and metabolic complications [75]. This phenotypic change correlates with reduced hepatic enzyme activity, improved histological liver steatosis and inflammation. Unfortunately, there is less certainty regarding the impact on fibrosis [75]. High-intensity interval exercise is able to improve plasmatic levels of triglyceride-rich VLDL1 particles and LDL cholesterol and insulin resistance and other CVD risk factors. Thus, it is strongly recommended together with a dietary approach (hypolipemic diet). In this regard, the Mediterranean diet and similar dietary approaches are gaining more and more attention from the scientific community [76].

#### 3.3.2. The Use of Nutraceuticals in MAFLD

The use of nutraceuticals in managing MAFLD should not be underestimated. They can be effective either alone or in combination with dietary and lifestyle changes [77]. In particular, the nutraceuticals reviewed are those with a proven significant improvement in hepatic steatosis. However, their usage has some issues: they can have rather low bioavailability and limited effectiveness. In fact, individual genetics can affect nutraceuticals’ absorption, storage, and excretion [31]. Nutraceuticals mainly target inflammation, glycemia and insulinemia, LDL-C, and blood pressure [78]. In MAFLD patients, they effectively address liver inflammation, steatosis, and insulin resistance [Table 3].

Silymarin is known for its antioxidant, anti-inflammatory, and antifibrotic properties. It comprises seven flavonolignans (silybin A, silybin B, isosilybin A, isosilybin B, silychristin, iso-silychristin, and silydianin) and one flavonoid (taxifolin). It has shown benefits in improving liver enzymes and reducing hepatic steatosis. In particular, several clinical studies on NAFLD have shown that silymarin can delay the progression of liver disease, alleviate symptoms, and enhance the quality of life of patients [31]. Silymarin acts as a scavenger of free radicals. For this reason, it prevents lipid peroxidation and protects enzyme systems associated with hepatic cellular damage. Thus, it reduces oxidative stress and cytotoxicity [79]. The accepted and tolerated dosage of silymarin in NAFLD and MAFLD studies is 140 mg three times a day. The dosage can reduce deranged liver enzyme levels [79]. For example, Torre et al. showed that 4 months of silymarin administration significantly reduced transaminases and gamma-glutamyl transferase levels [90]. Further, Lee et al. found similar significant results after only 1 month of silymarin treatment and, importantly, the reduction was maintained for more than 4 years [91].

Omega-3 Fatty acids reduce triglyceride levels and improve hepatic steatosis and insulin resistance [31]. Further, several trials have investigated the role of omega-3 in the treatment of NASH in men. However, the variability of results among the studies can be explained by differences of product administration and experimental design (e.g., formulation of omega-3, the duration and dosage of the supplements, the endpoints, and the measured outputs such as exercise, dietary changes, and the genetic or epigenetic background of the participants) [80]. Although no study has demonstrated significant improvements in key histological prognostic features (namely, fibrosis), most trials have reported a reduction of steatosis grade. One study using biopsies found no change in steatosis after 12 months of treatment with a synthetic Eicosapentaenoic acid (EPA) supplement (up to 2700 mg/day vs. placebo) [81].

Berberine lowers circulating lipids’ levels and enhances insulin sensitivity, contributing to improved metabolic profiles and a reduction in hepatic fat synthesis [31].

Curcumin is extracted from Curcuma Longa, has insulin-sensitizing effects and reduces liver inflammation and steatosis [31].

Coenzyme Q10 regulates adipokine levels and supports metabolic rebalancing in MAFLD [31].

Nigella Sativa with its active component, thymoquinone, possesses antioxidant and anti-inflammatory properties [31]. Specifically, it improves levels of transaminases, fasting glycemia, the lipid profile, the high-sensitivity C-reactive protein, and the degree of liver steatosis [84].

The combination of *Ascophyllum nodosum* and *Fucus vesiculosus* has been studied for its antioxidant, anti-inflammatory, and anti-cancer properties. These two types of brown algae enhance intestinal viscosity, slowing the absorption of cholesterol and inhibiting alfa (α)-amylase and α-glucosidase activity. The latter reduces sugar absorption. This combination significantly lowers insulin levels, blood glucose, the calculated HOMA-IR, and waist circumference. After six months of algae use, plasma HDL-C levels significantly rose in NAFLD subjects [85].

Vitamin E is a complex of tocopherols and tocotrienols extensively studied for the treatment of NASH due to its well-known antioxidant properties [86]. Most studies have focused on alfa (α)-tocopherol, obtaining inconsistent findings. Vitamin E (800 IU/day) and ursodeoxycholic acid (12–15 mg/kg) administered for two years, alone or in combination vs. a single/double placebo, demonstrated histological improvement in the combination group only [87]. Positive outcomes were also observed under E and C vitamin combined administration. More interestingly, in pediatric NASH patients, pioglitazone (belonging to thiazolidinediones, an insulin sensitizer used in type 2 diabetic patients) demonstrated significantly greater efficacy than vitamin E in reversing liver fibrosis. Indeed, fibrosis reduction was of 47% for pioglitazone vs. 36% for vitamin E vs. 21% for the placebo. Therefore, the data suggest that a high vitamin E dosage (precisely, 800 IU/day) can beneficially affect mild pediatric NASH patients with only a limited effect in adults [88,89].

#### 3.3.3. Pharmacological Treatment of Cardiometabolic Profile in MAFLD: The Crucial Role of Lowering Cholesterol Remedies

Pharmacologic MAFLD treatment should target liver steatosis, the metabolic disturbances associated with the condition and prevention of liver fibrosis development. The pharmacological treatment of MAFLD has evolved significantly in the last twenty years, with a wide range of agents now being considered for managing the disease and its associated metabolic complications.

**Figure 3:**
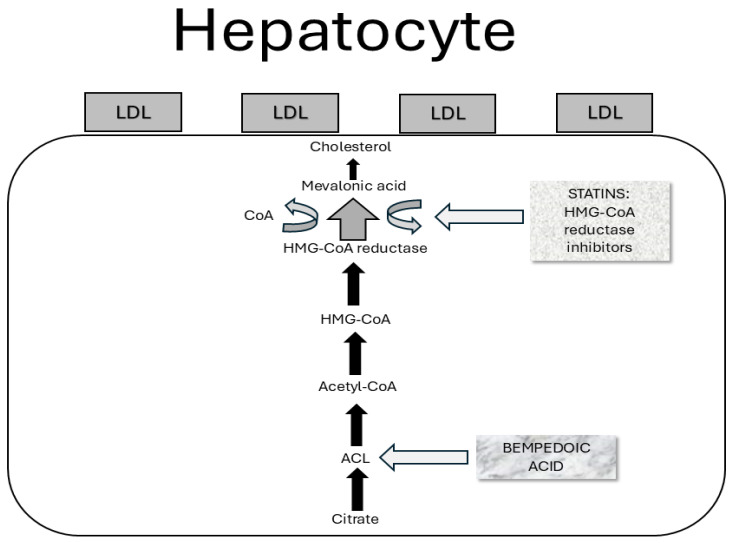
Mechanism of action of bempedoic acid. Bempedoic acid downregulates cholesterol biosynthesis by inhibiting ACL, a cytosolic enzyme that acts in the cholesterol synthesis chain on a phase prior to that of HMG-CoA reductase, the therapeutic target of statins.

*Statins* are commonly used in patients with MAFLD to manage hyperlipidemia and reduce cardiovascular risk. However, it is important to mention that their direct impact on liver histology is uncertain. In fact, statins can reduce liver fat content and improve liver enzymes but their effect on fibrosis is inconclusive. Epidemiological studies first supported the potential benefits of statins on liver function. These found statins’ use to be associated with a decreased risk of NAFLD/NASH and MAFLD/MASH diagnosis according to ultrasonography or histology usage [92,93]. Furthermore, statins’ use in diabetic patients was associated with a reduced risk for steatohepatitis and, even advanced liver fibrosis development [94]. In detail, atorvastatin reduces the expression of perilipin 5 in hepatocytes, contributes to increased lipolysis, and reduces triglyceride accumulation through protein kinase A phosphorylation [95]. Indeed, discontinuation of statin therapy remains a global issue in the frame of MAFLD treatment, mainly because of myalgia occurrence. Therefore, bempedoic acid (BA) usage has become more and more frequent. BA is an ATP citrate lyase inhibitor able to decrease the hepatic synthesis of cholesterol. In detail, it upregulates LDL receptor expression in the liver and clears circulating LDL-cholesterol from the bloodstream. Several randomized clinical trials showed a significant LDL level reduction (e.g., 17–28%) in statin-intolerant patients. Regarding the cholesterol biosynthesis cascade, ATP-citrate lyase (ACL) is an enzyme working two levels above HMG-CoA reductase. Indeed, its mechanism of action is similar to, but less efficient than, that of statins. Thus, BA mainly decreases the hepatic generation of cholesterol, upregulates the LDL receptor expression within the liver, and clears the circulating LDL-C from the systemic circulation [96] [Figure 3].

A promising class of drugs includes Pro-protein Convertase Subtilisin/Kexin type 9 inhibitors (PCSK9i) that can offer additional benefits in patients with MAFLD. Although clinical studies using PCSK9i are, at the time of writing, few, their results indicate high efficacy and safety. PCSK9 inhibitors may reduce liver steatosis, inflammation, and fibrosis [97]. Although PCSK9i should be prescribed in patients without a compromised liver function, Shafiq et al. showed that this treatment can lower hepatic transaminases’ levels [97]. Thus, PCSK9 inhibitors appear to have beneficial effects on patients with MAFLD. Notwithstanding, further research is necessary to gain more evidence for MAFLD treatment.

Although statins, the intestinal cholesterol transporter inhibitor (namely, ezetimibe) and PCSK9 inhibitors reduce serum levels of LDL-C, they do not act on Hypertriglyceridemia and HDL levels. The last two targets can be treated with fibrates, nicotinic acids, and n-3 polyunsaturated fatty acids. Fibrates have been shown to be PPARα agonists and can significantly lower triglycerides levels. However, they present some adverse effects, including an increase in creatinine levels and liver enzymes [65,97,98]. Intriguingly, several clinical trials demonstrate that Pemafibrate, the first Selective Peroxisome Proliferator-Activated Receptor Alpha Modulator (SPPARMα), can significantly lower liver enzymes and total bilirubin levels. The efficacy seems to be greater in more compromised patients [99]. These findings pave the road for its possible use in NAFLD/NASH patients that show more consistent evidence.

Antifibrotic therapies are gaining attention as they are showing promising preclinical studies. Agents that target TGF-beta and other fibrotic pathways could potentially reduce liver scarring and improve long-term outcomes in MAFLD patients [31]. To date, Resmetirom is the first Food and Drug Administration (FDA)-approved medication for the treatment of NASH/MASH. It is administered at either 80 mg or 100 mg per day. Resmetirom reduces liver fat accumulation by acting as an agonist of the thyroid hormone receptor (THR-β) [100]. In detail, the drug can provide NASH resolution (assessed by the NAFLD activity score) in 24.2% and 25.9% of patients treated with 80 and 100 mg, respectively, vs. 14.2% of those treated with a placebo (*p* < 0.001). Moreover, Resmetirom improves liver fibrosis (25.9% and 29.9%, after 80 mg and 100 mg, respectively, vs. 9.7% under placebo) (*p* < 0.001) in F2-F3 NASH patients [101].

Obeticholic acid (OCA), a semisynthetic derivative of the natural bile acid chenodeoxycholic acid, can improve insulin sensitivity and reduce biomarkers of liver fibrosis in NAFLD patients with type 2 diabetes mellitus [102]. However, we must bear in mind serious adverse events registered upon its use in non-cirrhotic patients with primary biliary cholangiopathy [103].

Antidiabetic medications are the most widely used pharmacological agents in the management of insulin resistance linked to MAFLD development. They can reduce hepatic glucose production and improve insulin resistance. Insulin sensitizers (e.g., pioglitazone and metformin) are recommended. Pioglitazone is recommended for patients diagnosed with MASH, while metformin has been found to markedly modify body composition and liver function in individuals with non-diabetic MAFLD [104]. Metformin reduces fat deposition and inhibits hepatic inflammation. This depends on enhanced phosphorylation of hepatic 5′ adenosine monophosphate-activated protein kinase (AMPK) and ACC and reduced lipogenic enzymes and proinflammatory cytokines [31].

Sodium-glucose cotransporter-2 (SGLT2) inhibitors improve liver enzymes and liver steatosis. For example, taking canagliflozin for 20 weeks delayed the onset of NASH and reduced liver enzymes, together with body weight [105]. Empagliflozin can improve both liver steatosis and fibrosis. It decreases transaminases in MAFLD patients with or without type 2 diabetes mellitus (T2DM) [106]. Furthermore, it decreases the hepatic expression of inflammatory cytokines (e.g., TNF-α, interleukin-6, and Monocyte Chemoattractant Protein-1 (MCP-1)) in NASH patients. When used in combination with linagliptin, a Dipeptidyl peptidase-4 (https://en.wikipedia.org/wiki/Dipeptidyl_peptidase-4 accessed on 1 May 2024) inhibitor (DPP-4i), it reduces mRNA expression of genes for fatty acid synthesis, collagen deposition, and the expression of Alpha Smooth Muscle Actin (αSMA). The latter is a biomarker of liver fibrosis [105,106]. Finally, empagliflozin attenuates inflammasome proteins’ expression and the triglyceride NOD-like receptor (NLR) family pyrin domain containing NLRP-3 activation in the liver [106].

Glucagon-Like Peptide-1 (GLP-1) Receptor Agonists slow down the progression of MAFLD through several mechanisms: reducing inflammation, improving insulin sensitivity, and mitigating oxidative stress. Moreover, they inhibit enzymes involved in hepatic lipogenesis, activate the autophagy/mitophagy pathway, and enhance the activity of enzymes responsible for beta (β)-oxidation. MAFLD patients seem to be their favorite target because of the peculiar downregulation of GLP-1 receptors [31]. In fact, almost 40% of patients treated with liraglutide showed steatohepatitis reversal at liver biopsy and improved glycemic profile, liver enzymes, and increased HDL concentration. This was consensual with weight loss of approximately 5 kg [107]. In line with these results, s.c. semaglutide once weekly shows liver enzymes’ normalization and reduced radiologic liver steatosis features in MASLD patients [108].

DPP-4i act via the suppression of the activity of DPP-4, leading to elevated incretin levels and reduced glucagon secretion. These increase insulin exocytosis, promote fatty acid oxidation in the liver, slow gastric emptying, and diminish hepatic glucose output [109]. Sitagliptin, a gliptin-based drug, can lower liver enzymes, reduce body weight, and reduce hepatocyte swelling in patients with concomitant diabetes and NASH [110].

## 4. Conclusions and Future Perspectives

In conclusion, the management of MAFLD requires a multifaceted approach, addressing all the metabolic components of the disease pathophysiology.

Solid evidence confirms that lifestyle modification, including correct nutrition combined with regular physical activity, has efficacy in reducing liver fat deposition and improving insulin sensitivity. Among the antioxidant substances available, high-dose Vitamin E has the most solid evidence, especially in pediatric NASH patients.

The use of pharmacological treatments such as statins, bempedoic acid, or PCSK9 inhibitors show promising results in MAFLD management and deserve further studies to confirm their capability to also revers liver fibrosis. In detail, statins can normalize liver function indexes but seem to not affect the liver fibrosis process. Indeed, they are a cornerstone in preventing fibrosis process in NAFLD/MAFLD subjects.

Newly released antifibrotic therapies show a promising impact on NASH/MASH patients for fibrosis reversal. More data are needed to confirm the first results.

Insulin sensitizers, such as Pioglitazone and metformin, can be used in diabetic and non-diabetic MASH patients, respectively, to reverse the disease course.

SGLT2 inhibitors and fibrates offer promising evidence for their use in treating dyslipidemia, insulin resistance, and inflammation in MAFLD.

GLP-1 receptor agonists have a very promising profile of action for liver fibrosis treatment in MAFLD and MASH patients, with special attention on body composition modification.

Dipeptidyl Peptidase-4 (DPP-4) inhibitors have a promising effect profile in MAFLD and NASH patients. Their potential usage can be considered in diabetic patients.

Finally, liver fat deposition, cholesterol synthesis, and transport until triglycerides’ extracellular storage in the liver are targets for the reviewed treatments. However, a personalized therapeutic approach cannot be overstated due to the multifaceted physiopathology of MAFLD. Future artificial-intelligence-powered therapeutic flow-charts are warranted to fit a personalized approach to every single patient with an MAFLD diagnosis, according to the disease staging.

## Figures and Tables

**Table 1:** Criteria for MAFLD diagnosis, based on the metabolic alterations provided [13].

Metabolic Alteration	Criteria
Waist Circumference	≥102 cm in Caucasian men, ≥88 cm in Caucasian women; ≥90 cm in Asian men, ≥80 cm in Asian women
Blood Pressure	≥130/85 mmHg or use of antihypertensive drugs
Plasma Triglycerides (TG)	≥150 mg/dL or use of TG-lowering drugs
Plasma High-Density Lipoprotein Cholesterol (HDL-C)	<40 mg/dL for men and <50 mg/dL for women, or use of lipid-lowering drugs
Fasting Plasma Glucose	Between 100 and 125 mg/dL or 2 h post-load glucose levels
Glucose Levels	Between 140 and 199 mg/dLor HbA1c between 5.7 and 6.4%
HOMA Index	Insulin resistance score ≥2.5
High-Sensitivity C-Reactive Protein (hs-CRP)	Levels > 2 mg/L

**Table 2:** Available indirect and direct indexes for monitoring disease activity and fibrosis in MAFLD.

Indirect Markers [11]	Direct Markers [22]	
	Collagen Synthesis/Degradation [23]	Pro-Inflammatory Molecules [24]
Aspartate amino Transferase (AST) [25]	PIIINP	TGF-Beta1
Alanine amino Transferase (AST) [25]	TIMP-1	GF-1
Platelet Count (PLT) [26]	TNF	CRP
Gamma Glutamil Transferase (GGT) [27]	MMP	Fibrinogen
Total Bilirubin [27]		Factor VIII
Alpha 2-macroglobulin and/or alpha 2 globulin [28]		PAI-1

**Table 3:** Nutraceuticals used in the treatment of MAFLD.

Nutraceutical	Key Properties	Benefits in MAFLD	References
Silymarin	Antioxidant, anti-inflammatory, antifibrotic	Improves liver enzymes and reduces hepatic steatosis	[31,79]
Omega-3 Fatty Acids	Reduces triglycerides, anti-inflammatory action	Lowers triglycerides, improves hepatic steatosis and insulin resistance	[80,81]
Berberine	Lipid-lowering, insulin-sensitizing	Enhances metabolic profile, reduces hepatic fat accumulation	[31,82]
Curcumin	Anti-inflammatory, insulin-sensitizing	Reduces liver inflammation, improves insulin sensitivity and hepatic steatosis	[31,83]
Coenzyme Q10	Anti-inflammatory, antioxidant action	Regulates adipokine levels support metabolic balance, reduces oxidative stress	[31,84]
Nigella Sativa	Antioxidant, anti-inflammatory (contains Thymoquinone) action	Improves liver enzyme, reduces inflammation and lowers cardiovascular risk markers	[85]
Brown Algae (*Ascophyllum nodosum* and *Fucus vesiculosus)*	antioxidant, anti-inflammatory, and anti-cancer properties	lowers insulin levels, Homeostatic Model Assessment for Insulin Resistance (HOMA-IR), blood glucose, and waist circumference	[85]
Vitamin E	Antioxidant action	improves liver function, particularly in pediatric NASH patients; can reduce liver inflammation	[86,87,88,89]

## Data Availability

All the data reviewed in the manuscript can be retrieved on the main medical database (e.g., PubMed, Medline) and on the website of the most important gastroenterology and hepatology international meetings (e.g., UEGW, DDW, AASLD, EASL).
